# The utility of hyperbaric oxygen therapy in post-transplant cyclophosphamide-induced hemorrhagic cystitis: a case report and review of the literature

**DOI:** 10.1186/s13256-020-02580-w

**Published:** 2021-01-04

**Authors:** Moayed Ibrahim, Kshama Bhyravabhotla, Basil Khalaf, Keith Van Meter, Nakhle S. Saba, Hana Safah, Francisco Socola

**Affiliations:** 1 Section of Hematology and Medical Oncology, Deming Department of Medicine, School of Medicine, Tulane University, 1430 Tulane Ave., Mailbox#8078, New Orleans, LA 70112-2699 USA; 2 Section of Pediatrics-Internal Medicine, Deming Department of Medicine, Tulane University, New Orleans, LA USA; 3 Section of Undersea and Hyperbaric Medicine, Department of Emergency Medicine, Louisiana State University, New Orleans, LA USA

**Keywords:** Post-transplant, Cyclophosphamide, Graft-versus-host disease prophylaxis, Acute myeloid leukemia, Hemorrhagic cystitis, Case report

## Abstract

**Background:**

To date, there are only a few case reports of cyclophosphamide (Cy)-induced hemorrhagic cystitis (HC) in adult or pediatric allogeneic stem cell transplant (SCT) patients treated successfully with hyperbaric oxygen (HBO). In all the reported cases, Cy was used as a part of the conditioning regimen, rather than post-transplant for graft-versus-host-disease (GVHD) prophylaxis. More recently, the risk of HC in allogeneic SCT is further increased by the widespread use of post-transplantation cyclophosphamide (PTCy) as a highly effective strategy for GVHD prophylaxis. This is the first case reported of PTCy-induced HC successfully treated with HBO to the best of our knowledge.

**Case presentation:**

In this article, we present a 58-year-old Caucasian male case of allogeneic SCT complicated by severe HC following PTCy, which was successfully treated with HBO, eliminating the need for cystectomy.

**Conclusion:**

HBO can be a safe, noninvasive, alternative treatment modality for PTCy-induced HC developing in allogeneic SCT patients.

## Background

Hemorrhagic cystitis (HC) affects 16.6% of patients treated with high dose cyclophosphamide (Cy) as part of the conditioning regimen in allogeneic stem cell transplant (SCT) patients [1]. More recently, the risk of HC is further increased by the widespread use of post-transplantation Cy (PTCy) as a highly effective strategy for graft-versus-host disease (GVHD) prophylaxis [2].

To date, there are only a few case reports of Cy-induced HC in adult or pediatric allogeneic SCT patients treated successfully with hyperbaric oxygen (HBO) [3–6], a review of which is summarized in Table 1. In all reported cases, Cy was used as a part of the conditioning regimen, not as PTCy.

**Table 1:** A review of studies of Cy-induced hemorrhagic cystitis in adult or pediatric allogeneic stem cell transplant patients treated successfully with hyperbaric oxygen

Study identifier	Study design	Inclusion criteria/study groups	Number of patients	Median age (year)	Gender (male: female)	Cyclophosphamide dose	Acute GVHD percentage	Infection percentage	Response rate (%)
K. Hattori *et al*. [11]	Case report	HC after allogeneic SCT in children	2	7	2:00	60 mg/kg/day for 2 days, 50 mg/kg/day for 4 days	0	0	100
S. Cesaro *et al*. [12]	Cross sectional	HC in pediatric patients after SCT	38	10.8	4:01	Not disclosed	20	71	84
M. Payandeh *et al*. [13]	Case report	HC after allogeneic SCT in AML patient	1	40	1:00	Not disclosed	100	0	100

In all reported cases, cyclophosphamide (Cy) was used as part of the conditioning regimen, not as post-transplantation Cy (PTCy). *HC* hemorrhagic cystitis, *SCT* stem cell transplant, *AML* acute myeloid leukemia, *GVHD* graft-versus-host disease

In this article, we present a case of allogeneic SCT complicated by severe HC following PTCy that was successfully treated with HBO, eliminating the need for cystectomy. To the best of our knowledge, this is the first case reported of PTCy-induced HC successfully treated with HBO.

## Case presentation

**Fig. 1:**
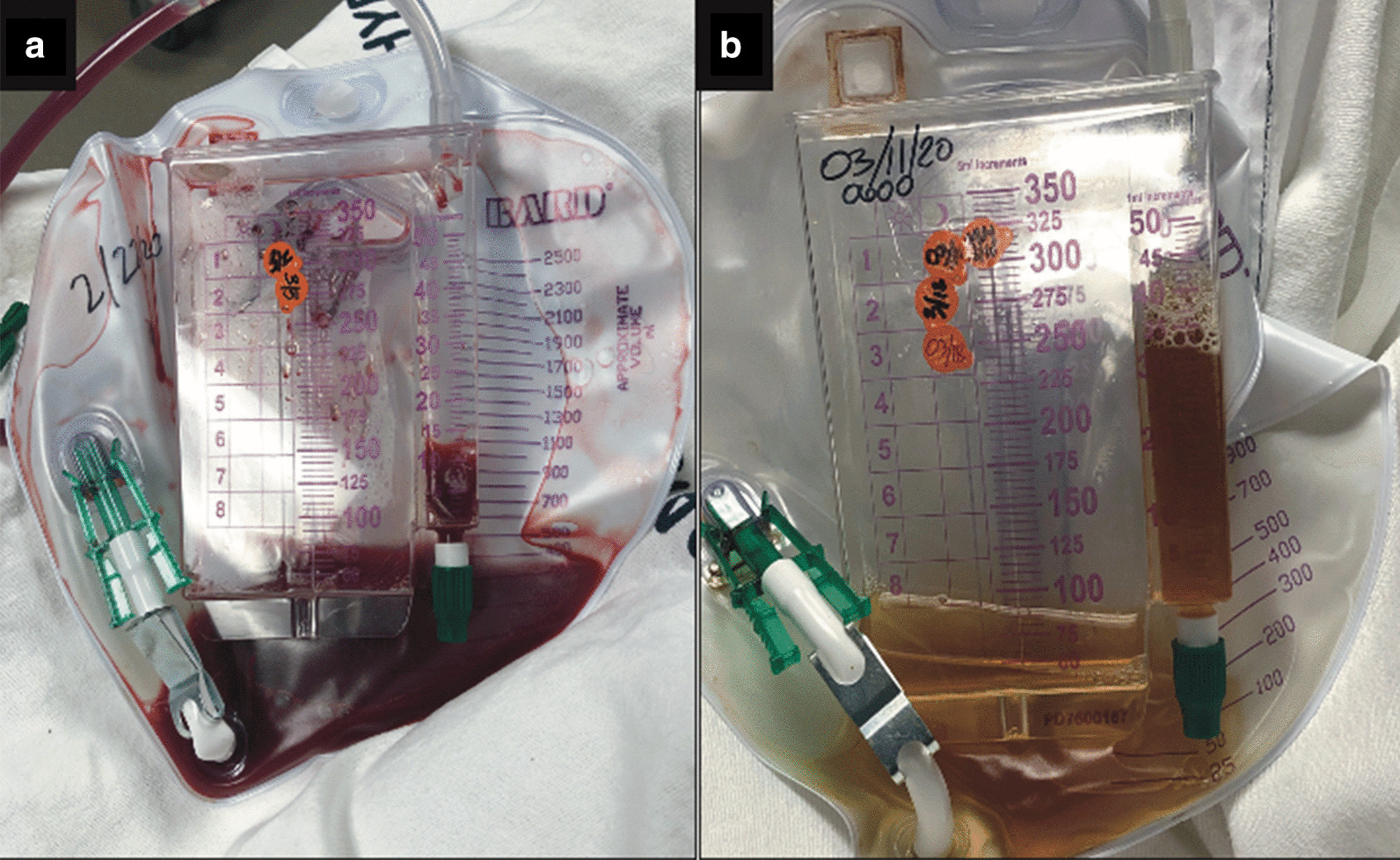
Comparison between the color of urine before (**a**) and after (**b**) completion of hyperbaric oxygen

A 58-year-old Caucasian male with acute myelogenous leukemia with myelodysplastic-related changes achieved a complete response following treatment with liposomal cytarabine-daunorubicin. The remission was consolidated with an allogeneic SCT from a 10/10 human leukocyte antigen (HLA)-matched unrelated donor, following a reduced-intensity conditioning regimen with fludarabine and melphalan. The graft-versus-host disease prophylaxis regimen consisted of post-transplant Cy (50 mg/kg on days +3 and +4 along with mesna at 50 mg/kg), tacrolimus (Tac) and mycophenolate mofetil (MMF). His transplant course was complicated with Epstein–Barr virus (EBV) viremia successfully treated with rituximab, delayed engraftment, and poor graft function. On day +70 post-SCT, he presented with acute kidney injury, severe gross hematuria with clots, and difficulty urinating. The patient had no history of pelvic irradiation, trauma, or urolithiasis. Physical exam was unremarkable apart from gross bloody urine with medium-sized clots (Fig. 1a). Urinalysis and culture were negative for bacteria. Viral studies in serum and urine were negative for cytomegalovirus, adenovirus, and BK virus. Regenerating islet-derived 3 alpha (REG3α) and suppression of tumorigenicity 2 (ST2) were elevated on admission, triggering the use of prednisone at 1 mg/kg for the possibility of bladder acute GVHD. MMF was stopped on day +35, and Tac was stopped due to the acute kidney injury. A bladder biopsy was not obtained due to the risk of worsening hematuria and perforation. Prednisone was discontinued 2 weeks later due to the lack of clinical benefit and absence of any other clinical signs of GVHD. Complete blood count showed severe anemia and thrombocytopenia requiring multiple blood and platelet transfusions. Computed tomography scanning of the pelvis obtained on admission showed a 1.7–cm round density at the posterior wall of the bladder consistent with a blood clot. As the patient complained of lower pelvic discomfort and difficulty urinating, an 18 Fr Foley catheter was inserted under continuous irrigation. A cystogram showed a large right-sided filling defect concerning for a large blood clot; this was followed by cystoscopy that revealed bleeding and friable bladder mucosa. Due to the severity of the hematuria and the need for multiple blood transfusions, the patient underwent bilateral urinary diversion using nephrostomy tubes. Aminocaproic acid infusion was also initiated with 4 g intravenous push followed by 1 g/hour, discontinued a week later due to lack of clinical benefit. The patient was next started on orally administered conjugated estrogen at 3.75 mg every 8 hours, consistent with a previously published report [7]. However, gross hematuria persisted. Following all these unsuccessful attempts at stopping the HC, the patient was started on daily (Monday through Friday) HBO treatment sessions with 100% oxygen at 2.4 atm for 110 minutes, with two 5-minute air breaks, on day +110 post-SCT. After 20 sessions, the frequency of required bladder irrigation decreased, and the hematuria resolved, with urine transitioning to clear yellow color (Fig. 1b). The patient completed the 40 planned sessions as the most commonly adopted HBO schedule. The Foley catheter was removed on day +139, and serial bladder scans performed confirmed the patient’s ability to void spontaneously. The patient’s transfusion requirements decreased significantly, and his urinalysis confirmed resolution of microscopic hematuria. Currently, the patient is on day +250 and remains without hematuria and transfusion-independent. Bone marrow biopsy at 6 months post-SCT confirmed disease remission with 100% donor chimerism (CD33 and T cells).

HBO rarely results in barotrauma in the middle ear, sinuses [8], or lungs [9], reversible myopia [8], seizures [10], or decompression sickness [11]. Our patient had none of these manifestations but reported initial worsening of his lower pelvic discomfort, described as pressure, which eventually resolved by the completion of HBO. The patient reported significant improvement in his quality of life upon resolution of HC. He also described HBO as tolerable and actually would recommend it to other patients if they had the same clinical challenge, that is, PTCy-induced HC.

## Discussion and conclusions

This case of HC following PTCy was refractory to commonly used measures, but fully recovered with HBO. The risk of HC directly correlates with the dose and cumulative amounts of cyclophosphamide [12]. Additional risk factors for HC in SCT recipients include male gender, age < 20 years, and acute GVHD [1].

Acrolein, a cyclophosphamide metabolite, is responsible for inducing the aseptic inflammatory process resulting in gross hematuria [13, 14]. Acrolein damages the urothelium by cleaving intracellular proteins and breaking DNA strands [15]. In addition, acrolein induces reactive oxygen and nitrogen species (ROS and RNS, respectively) [15]. ROS and RNS trigger proapoptotic pathways via producing superoxide radicals which break DNA cross-links, leading to cell death [15]. Mesna, a thiol compound that binds and neutralizes acrolein in the urine, is used as a preventive measure for HC [13, 14]. Unfortunately, mesna fails in 2% to 40% of cases [16], with no standard of care to further treat this complication, leading in most cases to partial or radical cystectomy.

Hyperbaric oxygen (HBO) is a well-established modality in treating radiation-induced but not Cy-induced HC [3]. HBO utilizes pressurized oxygen or air at pressures exceeding 2.8 to 3.0 atm as an adjunctive modality to treat various health conditions. HBO promotes wound healing via improved tissue oxygenation, thus reducing local hypoxia and ROS and RNS production [17].

HBO is not used in the treatment of BK-induced HC, in which reduction of immunosuppression and cidofovir administration could be a good option [18].

Our patient was initially treated with continuous bladder irrigation, urinary diversion, aminocaproic acid, conjugated estrogens, and transfusion support, without any clinical benefit. However, 40 sessions of HBO resulted in complete response. While HBO has been used in a few reported cases of Cy-induced HC in the setting of SCT, none has been reported as secondary to PTCy. With the adoption of PTCy as a new standard for GVHD prophylaxis [19], we anticipate that HC may become a more commonly encountered complication in allogeneic SCT patients. This risk might be further increased by the use of total-body irradiation and/or Cy as part of the conditioning regimen. Should this complication be encountered, HBO emerges as an acceptable, non-toxic, yet effective option that clinicians can rely on to treat HC. Ruling out other causes of hematuria such as BK virus infection, bacterial infections, tumor involvement, and urolithiasis should be completed prior to HBO. While Cy-induced HC remains a relatively uncommon complication, a prospective study is warranted as a standard tool to evaluate the efficacy of HBO in Cy- or PTCy-induced HC in the setting of allogeneic SCT.

## Data Availability

Not applicable.

## Abbreviations

Cy: Cyclophosphamide
PT: post-transplant
PTCy: Post-transplant cyclophosphamide
HC: Hemorrhagic cystitis
SCT: Stem cell transplant
HBO: Hyperbaric oxygen
GVHD: Graft-versus-host-disease
ROS: Reactive oxygen species
RNS: Reactive nitrogen species
